# Supplementary material for: Lactate dehydrogenase can be used for differential diagnosis to identify patients with severe polytrauma with or without chest injury—A retrospective study
Source: PLoS One. 2024 Aug 1;19(8):e0308228. doi: 10.1371/journal.pone.0308228 (PMC11293635; doi:10.1371/journal.pone.0308228)
Supplement: S1 Table — This table shows the results of the comparison between Group A and Group B for all biomarkers. (DOCX) [file pone.0308228.s001.docx]

| **Supplementary Table 1. All results of comparison of biochemical markers between patients with severe polytrauma without chest injury (Group A) and with chest injury (Group B)** | | | | |
| --- | --- | --- | --- | --- |
| **Variable** | | **A Group** | **B Group** | **P value** |
|  |  | **n=29** | **n=35** |  |
| HR (beats/min) | 0h | 69.04 ± 19.18 | 76.18 ± 13.77 | 0.157 |
|  | 8h | 70.04 ± 17.41 | 72.76 ± 13.97 | 0.524 |
|  | d1 | 71.54 ± 17.62 | 69.53 ± 14.22 | 0.645 |
|  | d2 | 73.84 ± 14.92 | 66.92 ± 12.48 | 0.101 |
|  | d3 | 74.28 ± 14.89 | 68.17 ± 9.47 | 0.112 |
|  | d4 | 74 ± 16.24 | 69.39 ± 10.43 | 0.282 |
|  | d5 | 73.44 ± 13.82 | 68.71 ± 9.95 | 0.235 |
|  | d6 | 77.86 ± 15.08 | 68.2 ± 10.44 | 0.034^*^ |
|  | d7 | 74.23 ± 16.05 | 72 ± 13.08 | 0.665 |
|  | d8 | 74.64 ± 12.86 | 71.67 ± 9.86 | 0.464 |
|  | d9 | 75.5 ± 12.57 | 75.28 ± 11.02 | 0.958 |
|  | d10 | 76.85 ± 15.54 | 70.94 ± 10.89 | 0.231 |
| RR (times/min) | 0h | 16.71 ± 4.04 | 16.75 ± 2.54 | 0.971 |
|  | 8h | 16.35 ± 3.55 | 18.23 ± 3.28 | 0.060 |
|  | d1 | 14.43 ± 3.56 | 17.41 ± 4.53 | 0.017^*^ |
|  | d2 | 14.54 ± 3.67 | 17.27 ± 8 | 0.255 |
|  | d3 | 15.58 ± 4.01 | 16.91 ± 6.41 | 0.521 |
|  | d4 | 16.73 ± 3 | 19.24 ± 14.15 | 0.568 |
|  | d5 | 17.4 ± 2.91 | 19.78 ± 6.25 | 0.183 |
|  | d6 | 17.44 ± 3.64 | 17.94 ± 4.66 | 0.781 |
|  | d7 | 17 ± 2.71 | 19.78 ± 5.2 | 0.195 |
|  | d8 | 17 ± 3.16 | 18.82 ± 6.9 | 0.487 |
|  | d9 | 18.86 ± 2.97 | 18.27 ± 7.05 | 0.835 |
|  | d10 | 19.29 ± 2.06 | 19.33 ± 5.54 | 0.977 |
| Age |  | 51.52 ± 21.65 | 50.57 ± 20.1 | 0.857 |
| Time in hospital (days) |  | 11.37 ± 13.76 | 10.44 ± 11.53 | 0.775 |
| ISS |  | 30.43 ± 9.28 | 30.88 ± 10.24 | 0.894 |
| GCS | 0h | 10.5 ± 5.72 | 7.5 ± 5.43 | 0.319 |
|  | 8h | 8.57 ± 6.11 | 10.14 ± 5.84 | 0.632 |
|  | d1 | 11.29 ± 5.3 | 8.12 ± 5.56 | 0.118 |
|  | d2 | 10.2 ± 6 | 8.1 ± 5.36 | 0.420 |
|  | d3 | 9 ± 5.64 | 8.5 ± 5.32 | 0.833 |
|  | d4 | 10.33 ± 5.72 | 8.5 ± 5.81 | 0.535 |
|  | d5 | 12.43 ± 4.43 | 8.73 ± 5.34 | 0.128 |
|  | d6 | 11.43 ± 5.77 | 9.27 ± 5.69 | 0.418 |
|  | d7 | 10.67 ± 5.96 | 10.38 ± 6.12 | 0.930 |
|  | d8 | 10.5 ± 5.86 | 10 ± 6.04 | 0.874 |
|  | d9 | 10.83 ± 6.08 | 9.44 ± 6.13 | 0.673 |
|  | d10 | 9.6 ± 6.07 | 10.44 ± 5.81 | 0.802 |
| SOFA | d1 | 7.05 ± 3.69 | 9.35 ± 3.55 | 0.041^*^ |
|  | d2 | 7.85 ± 3.08 | 9.56 ± 3 | 0.106 |
|  | d3 | 9.45 ± 3.59 | 9.17 ± 3.1 | 0.816 |
|  | d4 | 8 ± 3.1 | 8.52 ± 2.52 | 0.610 |
|  | d5 | 7.2 ± 2.04 | 9.32 ± 3.64 | 0.102 |
|  | d6 | 7.33 ± 3.28 | 9.32 ± 3.84 | 0.195 |
|  | d7 | 7.11 ± 3.02 | 7.63 ± 3.8 | 0.722 |
|  | d8 | 7 ± 2.83 | 7.72 ± 2.52 | 0.506 |
|  | d9 | 7.89 ± 3.92 | 8.06 ± 2.54 | 0.894 |
|  | d10 | 6.89 ± 3.89 | 7.56 ± 2.68 | 0.613 |
| presence of organ failure | | 3(29) | 5(35) | 0.635 |
| Red blood cell transfusion (U) | | 1.5 ± 4.28 | 3.59 ± 6.26 | 0.126 |
| Plasma transfusion (U) |  | 2.41 ± 5.63 | 3.68 ± 4.46 | 0.325 |
| Blood platelet transfusion (U) | | 0.1 ± 0.41 | 0.18 ± 0.72 | 0.629 |
| Urine Output (ml) | 0h | 671.11 ± 289.5 | 1034.29 ± 672.5 | 0.165 |
|  | 8h | 1026.44 ± 724.75 | 1194 ± 990.96 | 0.591 |
|  | d1 | 1545.83 ± 1240.93 | 2147.59 ± 926.1 | 0.054 |
|  | d2 | 2205.59 ± 1357.55 | 2406.74 ± 939.57 | 0.583 |
|  | d3 | 2357.33 ± 622.2 | 3102.17 ± 1172.67 | 0.030^*^ |
|  | d4 | 2690 ± 1115.95 | 2806.09 ± 863.6 | 0.725 |
|  | d5 | 2859.62 ± 935.98 | 2802.25 ± 1207.8 | 0.886 |
|  | d6 | 2336.43 ± 1051.51 | 2933.06 ± 1084.4 | 0.128 |
|  | d7 | 2691.67 ± 1202.71 | 2969.47 ± 908.92 | 0.470 |
|  | d8 | 2447.73 ± 780.77 | 3126.28 ± 1145.62 | 0.095 |
|  | d9 | 2750.91 ± 1030.04 | 3130 ± 840.8 | 0.296 |
|  | d10 | 2823.64 ± 1126.02 | 2646.67 ± 894.07 | 0.659 |
| Hb (g/dl) | 0h | 12.78 ± 1.65 | 12.77 ± 1.83 | 0.987 |
|  | 8h | 11.41 ± 1.93 | 11.46 ± 3.12 | 0.954 |
|  | d1 | 10.35 ± 1.94 | 10.21 ± 2.61 | 0.823 |
|  | d2 | 9.34 ± 1.94 | 9.51 ± 3.36 | 0.839 |
|  | d3 | 9.38 ± 2.12 | 9.14 ± 2.61 | 0.728 |
|  | d4 | 9.05 ± 2.05 | 8.59 ± 1.36 | 0.359 |
|  | d5 | 9.75 ± 2.24 | 8.67 ± 1.35 | 0.036^*^ |
|  | d6 | 9.48 ± 2.2 | 8.47 ± 1.17 | 0.096 |
|  | d7 | 9.11 ± 1.9 | 8.67 ± 1.44 | 0.397 |
|  | d8 | 8.78 ± 1.58 | 8.37 ± 0.79 | 0.363 |
|  | d9 | 8.74 ± 1.92 | 8.41 ± 1.01 | 0.551 |
|  | d10 | 8.29 ± 1.3 | 9.91 ± 6.59 | 0.372 |
| Leukocyte (U/nl) | 0h | 15.24 ± 6.73 | 15.17 ± 6.26 | 0.965 |
|  | 8h | 12.03 ± 4.45 | 12.83 ± 5.55 | 0.606 |
|  | d1 | 10.2 ± 3.56 | 9.9 ± 3.88 | 0.772 |
|  | d2 | 11.19 ± 3.58 | 9.64 ± 2.86 | 0.098 |
|  | d3 | 10.54 ± 3.85 | 8.01 ± 2.88 | 0.010^*^ |
|  | d4 | 8.94 ± 3.85 | 8.42 ± 4.38 | 0.675 |
|  | d5 | 8.93 ± 2.8 | 9.3 ± 4.58 | 0.747 |
|  | d6 | 9.29 ± 2.06 | 9.52 ± 3.52 | 0.813 |
|  | d7 | 9.94 ± 2.9 | 10.22 ± 3.75 | 0.803 |
|  | d8 | 10.94 ± 3.09 | 13.2 ± 5.75 | 0.126 |
|  | d9 | 11.46 ± 3.37 | 13.83 ± 6.23 | 0.138 |
|  | d10 | 12.17 ± 4.42 | 14.5 ± 4.55 | 0.134 |
| Platelet (U/nl) | 0h | 253.93 ± 61.51 | 235 ± 66.06 | 0.272 |
|  | 8h | 194.74 ± 62.05 | 184.96 ± 78.34 | 0.651 |
|  | d1 | 194.54 ± 61.64 | 171.83 ± 58.37 | 0.175 |
|  | d2 | 151.68 ± 49.25 | 138.29 ± 43.32 | 0.319 |
|  | d3 | 182.2 ± 81.78 | 139.26 ± 44.77 | 0.023^*^ |
|  | d4 | 191.88±65.82 | 151.79±53.69 | 0.012^*^ |
|  | d5 | 200.52 ± 66.65 | 174.58 ± 73.78 | 0.217 |
|  | d6 | 210.65 ± 64.91 | 203.96 ± 76.64 | 0.770 |
|  | d7 | 258 ± 93.05 | 251.38 ± 98.38 | 0.830 |
|  | d8 | 294.33 ± 77.38 | 306.57 ± 123.05 | 0.709 |
|  | d9 | 334.33 ± 99.13 | 346.13 ± 143.49 | 0.783 |
|  | d10 | 391.93 ± 124.05 | 396.83 ± 161.37 | 0.923 |
| Hematocrit (%) | 0h | 37.79 ± 4.34 | 40.1 ± 12.01 | 0.342 |
|  | 8h | 33.4 ± 6.04 | 31.7 ± 5.28 | 0.302 |
|  | d1 | 30.8 ± 5.76 | 30.93 ± 13.08 | 0.963 |
|  | d2 | 27.62 ± 6.03 | 28.93 ± 13.68 | 0.696 |
|  | d3 | 27.9 ± 6.49 | 28.57 ± 14.32 | 0.841 |
|  | d4 | 27.31 ± 6.37 | 28.44 ± 14.57 | 0.753 |
|  | d5 | 27.59 ± 5.4 | 28.04 ± 15.4 | 0.900 |
|  | d6 | 28.45 ± 5.95 | 26.06 ± 3.39 | 0.147 |
|  | d7 | 27.44 ± 5.29 | 26.59 ± 4.13 | 0.566 |
|  | d8 | 26.47 ± 4.15 | 25.85 ± 2.3 | 0.603 |
|  | d9 | 26.32 ± 5.37 | 25.97 ± 2.95 | 0.797 |
|  | d10 | 25.19 ± 3.9 | 26.31 ± 3.59 | 0.371 |
| CRP (mg/dl) | 0h | 0.37 ± 0.49 | 0.35 ± 0.4 | 0.902 |
|  | 8h | 0.56 ± 0.78 | 1.95 ± 2.26 | 0.096 |
|  | d1 | 5.77 ± 4.27 | 7.36 ± 6.23 | 0.485 |
|  | d2 | 17.06 ± 6.5 | 16.64 ± 7.03 | 0.852 |
|  | d3 | 15.49 ± 8.08 | 17.31 ± 6.93 | 0.439 |
|  | d4 | 12.96 ± 6.86 | 17.06 ± 8.47 | 0.123 |
|  | d5 | 13.89 ± 8.82 | 16.93 ± 8.42 | 0.271 |
|  | d6 | 10.52 ± 8.33 | 13.79 ± 7.59 | 0.118 |
|  | d7 | 11.85 ± 10.04 | 14.24 ± 7.49 | 0.400 |
|  | d8 | 13.13 ± 8.55 | 15.31 ± 6.19 | 0.402 |
|  | d9 | 16.03 ± 10.39 | 15.15 ± 7.89 | 0.794 |
|  | d10 | 15.52 ± 9.94 | 12.55 ± 7.86 | 0.368 |
| K^+^ | 0h | 4.03 ± 0.71 | 4.19 ± 0.48 | 0.291 |
|  | 8h | 4.18 ± 0.39 | 4.29 ± 0.47 | 0.447 |
|  | d1 | 4.34 ± 0.48 | 4.32 ± 0.41 | 0.864 |
|  | d2 | 4.15 ± 0.45 | 4.09 ± 0.58 | 0.718 |
|  | d3 | 4.18 ± 0.55 | 3.93 ± 0.36 | 0.102 |
|  | d4 | 4.23 ± 0.51 | 4.03 ± 0.34 | 0.137 |
|  | d5 | 4.18 ± 0.58 | 4.24 ± 0.35 | 0.711 |
|  | d6 | 4.43 ± 0.54 | 4.22 ± 0.45 | 0.224 |
|  | d7 | 4.28 ± 0.64 | 4.18 ± 0.39 | 0.612 |
|  | d8 | 4.47 ± 0.37 | 4.32 ± 0.29 | 0.213 |
|  | d9 | 4.39 ± 0.56 | 4.44 ± 0.51 | 0.792 |
|  | d10 | 4.42 ± 0.63 | 4.27 ± 0.49 | 0.298 |
| Ca^2+^ | 0h | 1.97 ± 0.47 | 1.79 ± 0.51 | 0.155 |
|  | 8h | 1.37 ± 0.39 | 1.24 ± 0.23 | 0.244 |
|  | d1 | 1.25 ± 0.29 | 1.17 ± 0.07 | 0.201 |
|  | d2 | 1.33 ± 0.38 | 1.23 ± 0.29 | 0.383 |
|  | d3 | 1.41 ± 0.43 | 1.24 ± 0.3 | 0.221 |
|  | d4 | 1.47 ± 0.49 | 1.34 ± 0.43 | 0.442 |
|  | d5 | 1.53 ± 0.51 | 1.24 ± 0.34 | 0.106 |
|  | d6 | 1.31 ± 0.38 | 1.22 ± 0.34 | 0.535 |
|  | d7 | 1.34 ± 0.45 | 1.34 ± 0.43 | 0.992 |
|  | d8 | 1.39 ± 0.48 | 1.2 ± 0.27 | 0.275 |
|  | d9 | 1.4 ± 0.45 | 1.3 ± 0.38 | 0.556 |
|  | d10 | 1.44 ± 0.46 | 1.38 ± 0.46 | 0.726 |
| Cholesterol (mg/dl) | 0h | 0.41 ± 0.15 | 0.38 ± 0.14 | 0.568 |
|  | 8h | 0.89 ± 0.65 | 0.75 ± 0.54 | 0.442 |
|  | d1 | 0.74 ± 0.38 | 0.76 ± 0.47 | 0.880 |
|  | d2 | 0.79 ± 0.71 | 0.74 ± 0.47 | 0.747 |
|  | d3 | 0.64 ± 0.62 | 0.71 ± 0.56 | 0.671 |
|  | d4 | 0.63 ± 0.58 | 0.74 ± 0.65 | 0.568 |
|  | d5 | 0.67 ± 0.63 | 0.84 ± 0.88 | 0.493 |
|  | d6 | 0.74 ± 0.8 | 0.99 ± 1.23 | 0.480 |
|  | d7 | 0.79 ± 1.02 | 0.78 ± 0.91 | 0.967 |
|  | d8 | 0.85 ± 0.9 | 0.76 ± 0.7 | 0.743 |
|  | d9 | 0.82 ± 1.07 | 0.8 ± 0.79 | 0.955 |
|  | d10 | 1.17 ± 1.59 | 0.86 ± 0.84 | 0.471 |
| GOT (U/l) | 0h | 77.36 ± 89.28 | 172.9 ± 249.34 | 0.061 |
|  | 8h | 73.61 ± 65.04 | 187.91 ± 252.14 | 0.015^*^ |
|  | d1 | 76.96 ± 70.85 | 198.15 ± 314.56 | 0.036^*^ |
|  | d2 | 106.16 ± 94.76 | 198.13 ± 324.97 | 0.237 |
|  | d3 | 94.53 ± 90.97 | 194.57 ± 336.81 | 0.132 |
|  | d4 | 74.74 ± 59.29 | 137.21 ± 203.91 | 0.136 |
|  | d5 | 60.47 ± 39.57 | 102.19 ± 110.97 | 0.145 |
|  | d6 | 64.6 ± 40.76 | 86.17 ± 69.55 | 0.284 |
|  | d7 | 52.16±29.52 | 82.62±64.42 | 0.019^*^ |
|  | d8 | 48.16±21.33 | 70.35±47.73 | 0.020^*^ |
|  | d9 | 66.86 ± 41.28 | 68.17 ± 53.53 | 0.938 |
|  | d10 | 80.83 ± 51.89 | 62.76 ± 38.6 | 0.263 |
| GPT (U/l) | 0h | 30.43 ± 12.09 | 175.83 ± 329.29 | 0.329 |
|  | 8h | 59.18 ± 79.94 | 151.58 ± 250.37 | 0.090 |
|  | d1 | 37±22.27 | 143.85±289.56 | 0.039^*^ |
|  | d2 | 46.05 ± 32.97 | 147.42 ± 308.36 | 0.079 |
|  | d3 | 53.21 ± 48.63 | 155.6 ± 326.41 | 0.101 |
|  | d4 | 49.47 ± 43.71 | 140.37 ± 274.69 | 0.102 |
|  | d5 | 47.94 ± 37.15 | 112.12 ± 188.64 | 0.103 |
|  | d6 | 52.33 ± 39.96 | 94.24 ± 131.49 | 0.239 |
|  | d7 | 43.64±35.71 | 81.65±87.42 | 0.027^*^ |
|  | d8 | 56.8 ± 40.06 | 82.87 ± 71.46 | 0.207 |
|  | d9 | 64.21 ± 47.2 | 71 ± 56.98 | 0.711 |
|  | d10 | 75.92 ± 59.62 | 65.33 ± 46.08 | 0.573 |
| Creatinine (mg/dl) | 0h | 0.93 ± 0.36 | 1 ± 0.32 | 0.401 |
|  | 8h | 0.91 ± 0.28 | 0.91 ± 0.28 | 0.962 |
|  | d1 | 0.98 ± 0.51 | 0.94 ± 0.26 | 0.696 |
|  | d2 | 0.99 ± 0.74 | 0.97 ± 0.44 | 0.912 |
|  | d3 | 0.84 ± 0.61 | 0.83 ± 0.38 | 0.953 |
|  | d4 | 0.84 ± 0.61 | 0.88 ± 0.39 | 0.772 |
|  | d5 | 0.87 ± 0.61 | 0.89 ± 0.47 | 0.859 |
|  | d6 | 0.86 ± 0.65 | 0.83 ± 0.45 | 0.847 |
|  | d7 | 0.93 ± 0.79 | 0.79 ± 0.39 | 0.426 |
|  | d8 | 0.88 ± 0.7 | 0.77 ± 0.39 | 0.526 |
|  | d9 | 0.84 ± 0.63 | 0.77 ± 0.36 | 0.692 |
|  | d10 | 0.81 ± 0.63 | 0.7 ± 0.33 | 0.539 |
| CK (U/l) | 0h | 692.41 ± 915.63 | 752.52 ± 652.38 | 0.777 |
|  | 8h | 1812.12 ± 1823.31 | 2284.14 ± 1860.17 | 0.407 |
|  | d1 | 2100.7 ± 2713.89 | 2952.84 ± 3230.15 | 0.333 |
|  | d2 | 2272.06 ± 2513.09 | 3466.37 ± 4280.35 | 0.288 |
|  | d3 | 1742.07 ± 1884.62 | 3059.67 ± 4719.41 | 0.306 |
|  | d4 | 1186.43 ± 1043.78 | 2268.29 ± 3179.95 | 0.225 |
|  | d5 | 884.23 ± 623.62 | 1585.68 ± 2076.09 | 0.244 |
|  | d6 | 943.64 ± 764.7 | 1209.74 ± 1485.3 | 0.582 |
|  | d7 | 605.64 ± 367.27 | 1293.48 ± 2102.68 | 0.125 |
|  | d8 | 416.55 ± 275.85 | 1047.68 ± 1608.72 | 0.209 |
|  | d9 | 507.27 ± 472.02 | 945.57 ± 1305.03 | 0.293 |
|  | d10 | 629.9 ± 681.08 | 725.2 ± 838.25 | 0.758 |
| CK-MB (U/l) | 0h | 85.71 ± 83.16 | 67.67 ± 37.78 | 0.569 |
|  | 8h | 95.88 ± 91.64 | 82.67 ± 47.42 | 0.676 |
|  | d1 | 90.63 ± 70.82 | 152.95 ± 121.04 | 0.046^*^ |
|  | d2 | 94.5 ± 82.73 | 99.89 ± 123.62 | 0.955 |
| pH | 0h | 7.32 ± 0.07 | 7.29 ± 0.09 | 0.180 |
|  | 8h | 7.37 ± 0.04 | 7.38 ± 0.06 | 0.532 |
|  | d1 | 7.4 ± 0.04 | 7.42 ± 0.05 | 0.111 |
|  | d2 | 7.43 ± 0.06 | 7.42 ± 0.05 | 0.497 |
|  | d3 | 7.44 ± 0.04 | 7.42 ± 0.05 | 0.129 |
|  | d4 | 7.43 ± 0.09 | 7.44 ± 0.04 | 0.727 |
|  | d5 | 7.43 ± 0.03 | 7.43 ± 0.04 | 0.892 |
|  | d6 | 7.42 ± 0.05 | 7.42 ± 0.05 | 0.920 |
|  | d7 | 7.44 ± 0.05 | 7.43 ± 0.05 | 0.588 |
|  | d8 | 7.44 ± 0.04 | 7.43 ± 0.03 | 0.364 |
|  | d9 | 7.45 ± 0.04 | 7.43 ± 0.07 | 0.280 |
|  | d10 | 7.44 ± 0.03 | 7.44 ± 0.05 | 0.902 |
| Lactate (mg/dl) | 0h | 37.47 ± 25.57 | 42.4 ± 34.09 | 0.550 |
|  | 8h | 26.93 ± 19.58 | 37.99 ± 22.89 | 0.063 |
|  | d1 | 23.88 ± 15.57 | 36.58 ± 26.03 | 0.034^*^ |
|  | d2 | 22.37 ± 16.95 | 21.83 ± 16.03 | 0.921 |
|  | d3 | 21.92 ± 16.66 | 17.3 ± 11.5 | 0.402 |
|  | d4 | 17.35 ± 9.53 | 15.26 ± 7.56 | 0.503 |
|  | d5 | 14.58 ± 4.36 | 15.06 ± 5.7 | 0.817 |
|  | d6 | 15.12 ± 5.03 | 15.1 ± 6.39 | 0.993 |
|  | d7 | 13.4 ± 4.23 | 12.7 ± 4.12 | 0.683 |
|  | d8 | 13.6 ± 4.69 | 13.45 ± 4.55 | 0.936 |
|  | d9 | 16.8 ± 7.58 | 12.49 ± 4.23 | 0.144 |
|  | d10 | 13.6 ± 5.98 | 11.93 ± 4.3 | 0.425 |
| LDH (U/l) | 0h | 358.29 ± 191.29 | 533.06 ± 247.23 | 0.004^*^ |
|  | 8h | 355.5 ± 155.26 | 449.08 ± 203.89 | 0.212 |
|  | d1 | 308.89 ± 72.03 | 466.17 ± 176.94 | 0.014^*^ |
|  | d2 | 252 ± 74.95 | 490.33 ± 406.45 | 0.448 |
|  | d3 | 839.75 ± 1211.87 | 414.42 ± 218.4 | 0.534 |
|  | d4 | 889 ± 1269.24 | 359.38 ± 116.3 | 0.466 |
|  | d5 | 1258.5 ± 1488.46 | 360.33 ± 89.35 | 0.550 |
|  | d6 | 906 ± 1056.05 | 348 ± 136.72 | 0.457 |
|  | d7 | 958 ± 954.59 | 430 ± 101.4 | 0.577 |
|  | d8 | 248.25 ± 43.29 | 396.6 ± 118.72 | 0.051 |
|  | d9 | 626.67 ± 420.3 | 477.5 ± 216.68 | 0.490 |
|  | d10 | 578.67 ± 356.35 | 407.33 ± 102.18 | 0.468 |
| ALP | 0h | 67.71 ± 19.82 | 66.12 ± 22.94 | 0.814 |
|  | 8h | 54.22 ± 12.16 | 54.69 ± 12.7 | 0.932 |
|  | d1 | 49 ± 13.67 | 50.27 ± 10.92 | 0.830 |
|  | d2 | 43.67 ± 11.93 | 53 ± 10.89 | 0.236 |
|  | d3 | 95.6 ± 76.15 | 60.64 ± 12.2 | 0.364 |
|  | d4 | 87.4 ± 32.04 | 75 ± 32.87 | 0.563 |
|  | d5 | 88.09 ± 57.36 | 77.55 ± 25.00 | 0.573 |
|  | d6 | 86.67 ± 42.19 | 98 ± 41.45 | 0.723 |
|  | d7 | 86.25 ± 53.21 | 77.65 ± 30.16 | 0.587 |
|  | d8 | 76.08 ± 40.36 | 83.54 ± 26.66 | 0.441 |
|  | d9 | 113.4 ± 30.98 | 96 ± 12.44 | 0.155 |
|  | d10 | 85.73 ± 63.72 | 83.72 ± 32.41 | 0.911 |
| PCO_2_ | 0h | 50.2 ± 8.69 | 52.32 ± 12.1 | 0.463 |
|  | 8h | 43.78 ± 5.99 | 42.75 ± 7.78 | 0.591 |
|  | d1 | 42.3 ± 4.86 | 40.61 ± 6.49 | 0.307 |
|  | d2 | 41.22 ± 6.28 | 44.4 ± 5.1 | 0.088 |
|  | d3 | 40.92 ± 5.74 | 43.24 ± 5.61 | 0.242 |
|  | d4 | 41.49 ± 5.43 | 41.17 ± 5.69 | 0.874 |
|  | d5 | 42.05 ± 4.32 | 40.99 ± 6.04 | 0.610 |
|  | d6 | 41.93 ± 6.36 | 43.19 ± 6.29 | 0.617 |
|  | d7 | 38.67 ± 6.83 | 41.67 ± 7.14 | 0.307 |
|  | d8 | 39.25 ± 4.34 | 40.8 ± 3.9 | 0.348 |
|  | d9 | 38.43 ± 3.49 | 39.9 ± 6.3 | 0.527 |
|  | d10 | 39.59 ± 4.89 | 39.75 ± 6.2 | 0.947 |
| HCO_3_^-^ | 0h | 25.92 ± 3.56 | 24.77 ± 3.27 | 0.211 |
|  | 8h | 25.25 ± 2.31 | 24.4 ± 2.9 | 0.244 |
|  | d1 | 28.82 ± 14.13 | 26.26 ± 3.21 | 0.348 |
|  | d2 | 26.94 ± 3.03 | 28.18 ± 3.31 | 0.244 |
|  | d3 | 27.39 ± 3.47 | 27.98 ± 3.37 | 0.629 |
|  | d4 | 28.48 ± 2.45 | 27.4 ± 3.59 | 0.309 |
|  | d5 | 27.83 ± 2.23 | 27.01 ± 3.05 | 0.440 |
|  | d6 | 26.98 ± 2.56 | 27.49 ± 3.1 | 0.659 |
|  | d7 | 26.22 ± 3.39 | 27.58 ± 4.34 | 0.421 |
|  | d8 | 26.85 ± 2.76 | 26.98 ± 2.55 | 0.900 |
|  | d9 | 26.92 ± 2.1 | 26.44 ± 1.92 | 0.563 |
|  | d10 | 26.53 ± 2.58 | 26.12 ± 2.73 | 0.714 |
| PaO_2_/FiO_2_ | 0h | 394.5 ± 242.4 | 356.89 ± 157.54 | 0.706 |
|  | 8h | 373.07 ± 110.33 | 308 ± 134.73 | 0.137 |
|  | d1 | 307.2 ± 93.98 | 235.84 ± 88.54 | 0.053 |
|  | d2 | 259 ± 99.7 | 245.61 ± 90.78 | 0.763 |
|  | d3 | 251.5 ± 102.1 | 245.47 ± 88.59 | 0.881 |
|  | d4 | 288.5 ± 89.25 | 239.89 ± 74.05 | 0.159 |
|  | d5 | 313.14 ± 89.02 | 259.44 ± 87.6 | 0.184 |
|  | d6 | 238.5 ± 75.66 | 234.38 ± 83.47 | 0.917 |
|  | d7 | 246 ± 31.65 | 248.94 ± 84.85 | 0.908 |
|  | d8 | 214.75 ± 35.4 | 244.69 ± 69.96 | 0.424 |
|  | d9 | 196.25 ± 37.93 | 269.07 ± 82.71 | 0.112 |
|  | d10 | 221.25 ± 22.54 | 269.08 ± 94.74 | 0.344 |
| INR | 0h | 1.16 ± 0.29 | 4.51 ± 17.15 | 0.315 |
|  | 8h | 1.2 ± 0.19 | 1.16 ± 0.15 | 0.438 |
|  | d1 | 1.19 ± 0.15 | 1.2 ± 0.17 | 0.877 |
|  | d2 | 1.25 ± 0.17 | 1.24 ± 0.18 | 0.786 |
|  | d3 | 1.13 ± 0.17 | 1.16 ± 0.12 | 0.511 |
|  | d4 | 1.08 ± 0.16 | 1.12 ± 0.11 | 0.291 |
|  | d5 | 1.08 ± 0.14 | 1.15 ± 0.2 | 0.239 |
|  | d6 | 1.08 ± 0.15 | 1.13 ± 0.15 | 0.371 |
|  | d7 | 1.08 ± 0.16 | 1.12 ± 0.11 | 0.349 |
|  | d8 | 1.1 ± 0.15 | 1.14 ± 0.12 | 0.390 |
|  | d9 | 1.09 ± 0.14 | 1.16 ± 0.1 | 0.084 |
|  | d10 | 1.08 ± 0.1 | 1.13 ± 0.09 | 0.114 |
| PTT (sec) | 0h | 25.99 ± 5.8 | 26.29 ± 4.32 | 0.831 |
|  | 8h | 27.34 ± 5.19 | 30.56 ± 12.22 | 0.273 |
|  | d1 | 29.9 ± 9.71 | 27.96 ± 4.33 | 0.331 |
|  | d2 | 31.72 ± 8.94 | 31.52 ± 19.1 | 0.967 |
|  | d3 | 28.4 ± 4.37 | 28.86 ± 4.16 | 0.708 |
|  | d4 | 29.51 ± 5.36 | 29.88 ± 7.74 | 0.862 |
|  | d5 | 29.47 ± 7.07 | 29.3 ± 5.42 | 0.926 |
|  | d6 | 29.93 ± 6.69 | 30.28 ± 8.64 | 0.891 |
|  | d7 | 30.26 ± 7.75 | 28.8 ± 4.41 | 0.446 |
|  | d8 | 31.17 ± 8.07 | 27.91 ± 5.21 | 0.207 |
|  | d9 | 28.05 ± 4.99 | 29.94 ± 12.47 | 0.583 |
|  | d10 | 29.42 ± 3.97 | 29.51 ± 12.23 | 0.980 |
| *: P-value<0.05. Data shown as mean ± standard deviation. 0h: on admission; 8h: 8 hours post admission; dx: x days post admission.  HR: Heart rate; RR: Respiration rate; ISS: Injury Severity Score; GCS: Glasgow Coma Scale; SOFA: Sequential Organ Failure Assessment; Hb: Hemoglobin; CRP: C-reactive protein; K^+^: Potassium ions; Ca^2+^: Calcium ions; GOT: Glutamate oxaloacetate aminotransferase; GPT: Glutamate-pyruvate transaminase; CK: Creatine kinase; CK-MB: Creatine Kinase-MB; LDH: Lactate dehydrogenase; ALP: Alkaline phosphatase; PO_2_: Oxygen partial pressure; PCO_2_: Carbon dioxide partial pressure; FiO2: Fraction of inspiration Oxygen; HCO_3_^-^: Bicarbonate; PPT: Partial prothrombin time; INR: International normalized ratio. | | | | |
