# Supplementary material for: Lactate dehydrogenase can be used for differential diagnosis to identify patients with severe polytrauma with or without chest injury—A retrospective study
Source: PLoS One. 2024 Aug 1;19(8):e0308228. doi: 10.1371/journal.pone.0308228 (PMC11293635; doi:10.1371/journal.pone.0308228)
Supplement: S2 Table — This table delineates the findings from an analysis examining the correlation of all variables with the presence or absence of concurrent chest injuries in individuals suffering from severe polytrauma, wherein the presence of such injuries is denoted as a positive outcome. (DOCX) [file pone.0308228.s002.docx]

| **Supplementary Table 2. All results of the correlation analysis of various biochemical parameters with chest injury in polytrauma patients** | | | |
| --- | --- | --- | --- |
| **Variable** | | **r** | **P value** |
|  |  | **n=64** |  |
| HR (beats/min) | 0h | 0.212 | 0.157 |
|  | 8h | 0.088 | 0.524 |
|  | d1 | -0.064 | 0.645 |
|  | d2 | -0.250 | 0.101 |
|  | d3 | -0.249 | 0.112 |
|  | d4 | -0.174 | 0.282 |
|  | d5 | -0.200 | 0.235 |
|  | d6 | -0.364 | 0.034* |
|  | d7 | -0.078 | 0.665 |
|  | d8 | -0.134 | 0.464 |
|  | d9 | -0.010 | 0.958 |
|  | d10 | -0.225 | 0.231 |
| RR (times/min) | 0h | 0.007 | 0.971 |
|  | 8h | 0.271 | 0.060 |
|  | d1 | 0.377 | 0.010* |
|  | d2 | 0.198 | 0.255 |
|  | d3 | 0.114 | 0.521 |
|  | d4 | 0.105 | 0.568 |
|  | d5 | 0.216 | 0.269 |
|  | d6 | 0.056 | 0.781 |
|  | d7 | 0.268 | 0.195 |
|  | d8 | 0.146 | 0.487 |
|  | d9 | -0.047 | 0.835 |
|  | d10 | 0.005 | 0.983 |
| Age |  | -0.023 | 0.857 |
| Time in hospital (days) |  | -0.037 | 0.775 |
| ISS |  | 0.022 | 0.894 |
| GCS | 0h | -0.266 | 0.319 |
|  | 8h | 0.141 | 0.632 |
|  | d1 | -0.287 | 0.018* |
|  | d2 | -0.191 | 0.420 |
|  | d3 | -0.048 | 0.833 |
|  | d4 | -0.157 | 0.535 |
|  | d5 | -0.335 | 0.128 |
|  | d6 | -0.182 | 0.418 |
|  | d7 | -0.026 | 0.930 |
|  | d8 | -0.043 | 0.874 |
|  | d9 | -0.119 | 0.673 |
|  | d10 | 0.074 | 0.802 |
| SOFA | d1 | 0.306 | 0.041* |
|  | d2 | 0.266 | 0.106 |
|  | d3 | -0.041 | 0.816 |
|  | d4 | 0.094 | 0.610 |
|  | d5 | 0.310 | 0.102 |
|  | d6 | 0.253 | 0.195 |
|  | d7 | 0.070 | 0.722 |
|  | d8 | 0.134 | 0.506 |
|  | d9 | 0.027 | 0.894 |
|  | d10 | 0.106 | 0.613 |
| presence of organ failure | | 0.059 | 0.641 |
| Red blood cell transfusion (U) | | 0.190 | 0.139 |
| Plasma transfusion (U) |  | 0.126 | 0.325 |
| Blood platelet transfusion (U) | | 0.062 | 0.629 |
| Urine Output (ml) | 0h | 0.365 | 0.165 |
|  | 8h | 0.100 | 0.579 |
|  | d1 | 0.272 | 0.054 |
|  | d2 | 0.090 | 0.583 |
|  | d3 | 0.352 | 0.030* |
|  | d4 | 0.060 | 0.725 |
|  | d5 | -0.026 | 0.886 |
|  | d6 | 0.275 | 0.128 |
|  | d7 | 0.135 | 0.470 |
|  | d8 | 0.316 | 0.095 |
|  | d9 | 0.205 | 0.296 |
|  | d10 | -0.091 | 0.659 |
| Hb (g/dl) | 0h | -0.002 | 0.987 |
|  | 8h | 0.008 | 0.954 |
|  | d1 | -0.031 | 0.823 |
|  | d2 | 0.029 | 0.839 |
|  | d3 | -0.050 | 0.728 |
|  | d4 | -0.135 | 0.359 |
|  | d5 | -0.313 | 0.032* |
|  | d6 | -0.293 | 0.060 |
|  | d7 | -0.134 | 0.397 |
|  | d8 | -0.174 | 0.295 |
|  | d9 | -0.114 | 0.497 |
|  | d10 | 0.147 | 0.372 |
| Leukocyte (U/nl) | 0h | -0.006 | 0.965 |
|  | 8h | 0.077 | 0.606 |
|  | d1 | -0.040 | 0.772 |
|  | d2 | -0.236 | 0.098 |
|  | d3 | -0.358 | 0.010* |
|  | d4 | -0.062 | 0.675 |
|  | d5 | 0.048 | 0.747 |
|  | d6 | 0.037 | 0.813 |
|  | d7 | 0.040 | 0.803 |
|  | d8 | 0.226 | 0.173 |
|  | d9 | 0.219 | 0.186 |
|  | d10 | 0.248 | 0.134 |
| Platelet (U/nl) | 0h | -0.149 | 0.272 |
|  | 8h | -0.068 | 0.651 |
|  | d1 | -0.189 | 0.175 |
|  | d2 | -0.144 | 0.319 |
|  | d3 | -0.273 | 0.053 |
|  | d4 | -0.247 | 0.091 |
|  | d5 | -0.183 | 0.217 |
|  | d6 | -0.047 | 0.770 |
|  | d7 | -0.034 | 0.830 |
|  | d8 | 0.057 | 0.734 |
|  | d9 | 0.046 | 0.783 |
|  | d10 | 0.016 | 0.923 |
| Hematocrit (%) | 0h | 0.128 | 0.342 |
|  | 8h | -0.150 | 0.302 |
|  | d1 | 0.006 | 0.963 |
|  | d2 | 0.057 | 0.696 |
|  | d3 | 0.029 | 0.841 |
|  | d4 | 0.047 | 0.753 |
|  | d5 | 0.019 | 0.900 |
|  | d6 | -0.254 | 0.105 |
|  | d7 | -0.091 | 0.566 |
|  | d8 | -0.098 | 0.557 |
|  | d9 | -0.043 | 0.797 |
|  | d10 | 0.149 | 0.371 |
| CRP (mg/dl) | 0h | -0.020 | 0.902 |
|  | 8h | 0.382 | 0.118 |
|  | d1 | 0.143 | 0.485 |
|  | d2 | -0.032 | 0.852 |
|  | d3 | 0.123 | 0.439 |
|  | d4 | 0.258 | 0.123 |
|  | d5 | 0.178 | 0.271 |
|  | d6 | 0.392 | 0.015* |
|  | d7 | 0.137 | 0.400 |
|  | d8 | 0.151 | 0.402 |
|  | d9 | -0.050 | 0.794 |
|  | d10 | -0.168 | 0.368 |
| K^+^ | 0h | 0.135 | 0.291 |
|  | 8h | 0.125 | 0.447 |
|  | d1 | -0.027 | 0.864 |
|  | d2 | -0.059 | 0.718 |
|  | d3 | -0.273 | 0.084 |
|  | d4 | -0.233 | 0.137 |
|  | d5 | 0.062 | 0.711 |
|  | d6 | -0.208 | 0.224 |
|  | d7 | -0.102 | 0.572 |
|  | d8 | -0.226 | 0.213 |
|  | d9 | 0.051 | 0.792 |
|  | d10 | -0.397 | 0.036* |
| Ca^2+^ | 0h | -0.183 | 0.155 |
|  | 8h | -0.212 | 0.214 |
|  | d1 | -0.229 | 0.201 |
|  | d2 | -0.157 | 0.383 |
|  | d3 | -0.240 | 0.187 |
|  | d4 | -0.146 | 0.442 |
|  | d5 | -0.333 | 0.083 |
|  | d6 | -0.127 | 0.535 |
|  | d7 | -0.002 | 0.992 |
|  | d8 | -0.245 | 0.239 |
|  | d9 | -0.123 | 0.556 |
|  | d10 | -0.072 | 0.726 |
| Cholesterol (mg/dl) | 0h | -0.089 | 0.568 |
|  | 8h | -0.118 | 0.442 |
|  | d1 | 0.021 | 0.880 |
|  | d2 | -0.047 | 0.747 |
|  | d3 | 0.063 | 0.671 |
|  | d4 | 0.087 | 0.568 |
|  | d5 | 0.107 | 0.493 |
|  | d6 | 0.117 | 0.480 |
|  | d7 | -0.007 | 0.967 |
|  | d8 | -0.055 | 0.743 |
|  | d9 | -0.010 | 0.955 |
|  | d10 | -0.130 | 0.471 |
| GOT (U/l) | 0h | 0.250 | 0.061 |
|  | 8h | 0.264 | 0.045* |
|  | d1 | 0.281 | 0.048* |
|  | d2 | 0.170 | 0.237 |
|  | d3 | 0.181 | 0.213 |
|  | d4 | 0.190 | 0.202 |
|  | d5 | 0.226 | 0.145 |
|  | d6 | 0.176 | 0.284 |
|  | d7 | 0.233 | 0.042* |
|  | d8 | 0.271 | 0.049* |
|  | d9 | 0.013 | 0.938 |
|  | d10 | -0.201 | 0.263 |
| GPT (U/l) | 0h | 0.334 | 0.264 |
|  | 8h | 0.223 | 0.150 |
|  | d1 | 0.251 | 0.042* |
|  | d2 | 0.204 | 0.151 |
|  | d3 | 0.194 | 0.183 |
|  | d4 | 0.210 | 0.161 |
|  | d5 | 0.211 | 0.175 |
|  | d6 | 0.190 | 0.239 |
|  | d7 | 0.216 | 0.048* |
|  | d8 | 0.209 | 0.207 |
|  | d9 | 0.063 | 0.711 |
|  | d10 | -0.102 | 0.573 |
| Creatinine (mg/dl) | 0h | 0.113 | 0.401 |
|  | 8h | -0.007 | 0.962 |
|  | d1 | -0.056 | 0.696 |
|  | d2 | -0.016 | 0.912 |
|  | d3 | -0.009 | 0.953 |
|  | d4 | 0.043 | 0.772 |
|  | d5 | 0.027 | 0.859 |
|  | d6 | -0.031 | 0.847 |
|  | d7 | -0.126 | 0.426 |
|  | d8 | -0.106 | 0.526 |
|  | d9 | -0.068 | 0.692 |
|  | d10 | -0.111 | 0.539 |
| CK (U/l) | 0h | 0.039 | 0.777 |
|  | 8h | 0.125 | 0.407 |
|  | d1 | 0.138 | 0.333 |
|  | d2 | 0.157 | 0.288 |
|  | d3 | 0.156 | 0.306 |
|  | d4 | 0.191 | 0.225 |
|  | d5 | 0.194 | 0.244 |
|  | d6 | 0.098 | 0.582 |
|  | d7 | 0.180 | 0.292 |
|  | d8 | 0.224 | 0.209 |
|  | d9 | 0.192 | 0.293 |
|  | d10 | 0.059 | 0.758 |
| CK-MB (U/l) | 0h | -0.154 | 0.569 |
|  | 8h | -0.100 | 0.676 |
|  | d1 | 0.383 | 0.023* |
|  | d2 | 0.019 | 0.955 |
| pH | 0h | -0.182 | 0.180 |
|  | 8h | 0.089 | 0.532 |
|  | d1 | 0.228 | 0.111 |
|  | d2 | -0.114 | 0.497 |
|  | d3 | -0.262 | 0.129 |
|  | d4 | 0.064 | 0.727 |
|  | d5 | -0.026 | 0.892 |
|  | d6 | -0.020 | 0.920 |
|  | d7 | -0.109 | 0.588 |
|  | d8 | -0.182 | 0.364 |
|  | d9 | -0.225 | 0.280 |
|  | d10 | -0.026 | 0.902 |
| Lactate (mg/dl) | 0h | 0.083 | 0.550 |
|  | 8h | 0.288 | 0.053 |
|  | d1 | 0.305 | 0.033* |
|  | d2 | -0.017 | 0.921 |
|  | d3 | -0.166 | 0.341 |
|  | d4 | -0.123 | 0.503 |
|  | d5 | 0.045 | 0.817 |
|  | d6 | -0.002 | 0.993 |
|  | d7 | -0.082 | 0.683 |
|  | d8 | -0.016 | 0.936 |
|  | d9 | -0.358 | 0.079 |
|  | d10 | -0.167 | 0.425 |
| LDH (U/l) | 0h | 0.371 | 0.004* |
|  | 8h | 0.258 | 0.212 |
|  | d1 | 0.498 | 0.022* |
|  | d2 | 0.256 | 0.448 |
|  | d3 | -0.315 | 0.235 |
|  | d4 | -0.363 | 0.246 |
|  | d5 | -0.345 | 0.352 |
|  | d6 | -0.413 | 0.415 |
|  | d7 | -0.543 | 0.208 |
|  | d8 | 0.664 | 0.051 |
|  | d9 | -0.265 | 0.490 |
|  | d10 | -0.372 | 0.468 |
| ALP | 0h | -0.038 | 0.814 |
|  | 8h | 0.019 | 0.932 |
|  | d1 | 0.055 | 0.830 |
|  | d2 | 0.370 | 0.236 |
|  | d3 | -0.381 | 0.145 |
|  | d4 | -0.209 | 0.563 |
|  | d5 | -0.132 | 0.481 |
|  | d6 | -0.015 | 0.966 |
|  | d7 | -0.106 | 0.584 |
|  | d8 | 0.111 | 0.041* |
|  | d9 | -0.401 | 0.155 |
|  | d10 | -0.022 | 0.911 |
| PCO_2_ | 0h | 0.102 | 0.462 |
|  | 8h | -0.075 | 0.591 |
|  | d1 | -0.145 | 0.307 |
|  | d2 | 0.273 | 0.088 |
|  | d3 | 0.197 | 0.242 |
|  | d4 | -0.028 | 0.874 |
|  | d5 | -0.094 | 0.610 |
|  | d6 | 0.099 | 0.617 |
|  | d7 | 0.204 | 0.307 |
|  | d8 | 0.188 | 0.348 |
|  | d9 | 0.133 | 0.527 |
|  | d10 | 0.014 | 0.947 |
| HCO_3_^-^ | 0h | -0.170 | 0.211 |
|  | 8h | -0.163 | 0.244 |
|  | d1 | -0.133 | 0.348 |
|  | d2 | 0.189 | 0.244 |
|  | d3 | 0.083 | 0.629 |
|  | d4 | -0.162 | 0.361 |
|  | d5 | -0.144 | 0.440 |
|  | d6 | 0.087 | 0.659 |
|  | d7 | 0.161 | 0.421 |
|  | d8 | 0.025 | 0.900 |
|  | d9 | -0.121 | 0.563 |
|  | d10 | -0.077 | 0.714 |
| PaO_2_/FiO_2_ | 0h | -0.099 | 0.706 |
|  | 8h | -0.256 | 0.137 |
|  | d1 | -0.362 | 0.053 |
|  | d2 | -0.065 | 0.763 |
|  | d3 | -0.032 | 0.881 |
|  | d4 | -0.284 | 0.159 |
|  | d5 | -0.275 | 0.184 |
|  | d6 | -0.024 | 0.917 |
|  | d7 | 0.017 | 0.941 |
|  | d8 | 0.189 | 0.424 |
|  | d9 | 0.388 | 0.112 |
|  | d10 | 0.253 | 0.344 |
| INR | 0h | 0.138 | 0.315 |
|  | 8h | -0.116 | 0.438 |
|  | d1 | 0.022 | 0.877 |
|  | d2 | -0.040 | 0.786 |
|  | d3 | 0.097 | 0.511 |
|  | d4 | 0.159 | 0.291 |
|  | d5 | 0.183 | 0.239 |
|  | d6 | 0.145 | 0.371 |
|  | d7 | 0.150 | 0.349 |
|  | d8 | 0.150 | 0.390 |
|  | d9 | 0.288 | 0.084 |
|  | d10 | 0.272 | 0.114 |
| PTT (sec) | 0h | 0.029 | 0.831 |
|  | 8h | 0.161 | 0.273 |
|  | d1 | -0.135 | 0.331 |
|  | d2 | -0.006 | 0.967 |
|  | d3 | 0.055 | 0.708 |
|  | d4 | 0.026 | 0.862 |
|  | d5 | -0.015 | 0.926 |
|  | d6 | 0.022 | 0.891 |
|  | d7 | -0.122 | 0.446 |
|  | d8 | -0.245 | 0.149 |
|  | d9 | 0.093 | 0.583 |
|  | d10 | 0.004 | 0.980 |
| *: P-value<0.05; r: Pearson correlation coefficient 0h: on admission; 8h: 8 hours post admission; dx: x days post admission.  HR: Heart rate; RR: Respiration rate; ISS: Injury Severity Score; GCS: Glasgow Coma Scale; SOFA: Sequential Organ Failure Assessment; Hb: Hemoglobin; CRP: C-reactive protein; K^+^: Potassium ions; Ca^2+^: Calcium ions; GOT: Glutamate oxaloacetate aminotransferase; GPT: Glutamate-pyruvate transaminase; CK: Creatine kinase; CK-MB: Creatine Kinase-MB; LDH: Lactate dehydrogenase; ALP: Alkaline phosphatase; PO_2_: Oxygen partial pressure; PCO_2_: Carbon dioxide partial pressure; FiO_2_: Fraction of inspiration Oxygen; HCO_3_^-^: Bicarbonate; PPT: Partial prothrombin time; INR: International normalized ratio. | | | |
