# Supplementary material for: Lactate dehydrogenase can be used for differential diagnosis to identify patients with severe polytrauma with or without chest injury—A retrospective study
Source: PLoS One. 2024 Aug 1;19(8):e0308228. doi: 10.1371/journal.pone.0308228 (PMC11293635; doi:10.1371/journal.pone.0308228)
Supplement: Attachment — Submitted filename: Response to Reviewers.docx [file pone.0308228.s003.docx]

Dear Editor and Reviewers,

Thank you very much for your time and effort in reviewing our manuscript titled " Lactate dehydrogenase can be used for differential diagnosis to identify patients with severe polytrauma with or without chest injury—a retrospective study" We greatly appreciate your valuable comments and suggestions. The feedback provided has been instrumental in enhancing the quality and clarity of our manuscript. We have carefully considered each point raised and have made corresponding revisions to address these concerns.

We are resubmitting our manuscript after incorporating the changes suggested by you and hope that the revised manuscript meets the esteemed standards of PlosOne. Below, we provide detailed responses to each of the comments and outline the modifications we have made to the manuscript. Thank you once again for the opportunity to improve our work.

Response to Reviewer #1

1. In response to the first and second comments. Thank you for your suggestion, as you said, there is a descriptive problem with the definitions here, now we have defined polytrauma, severe trauma and severe polytrauma more accurately based on your suggestions and have added references to support the basis of our definitions. Changes are made on lines 79 to 83 of the manuscript.
2. In response to the third comment. Yes, the threshold for patients having a chest injury is chest AIS ≥1. Thank you very much for the reminder, we have added a description on line 83 of the manuscript.
3. In response to the fourth comment.

In this study, we analyzed patients by indicators such as age, gender, and Injury Severity Score (ISS), observing no significant differences in outcomes among the groups; detailed results are provided in the Supporting Materials. Our patient cohort consisted of individuals suffering from severe polytrauma. We categorized these patients based on the presence or absence of chest injuries. It is important to note that while other parenchymal organ injuries such as liver, kidney, and spleen were identified, their incidence did not differ between our two groups of patients (6/29 vs. 8/35, P=0.835). Our findings revealed no differences in ISS scores between groups, indicating a uniform severity of trauma across the cohort. This suggests that the presence of chest injuries does not correlate with a higher overall injury severity under the conditions of this study.

Moreover, we addressed potential confounding factors using a multifactorial logistic regression model. This analysis helped to clarify the impact of variables that appeared significant in univariate analyses, such as glutamic-pyruvic transaminase (GPT) and lactate levels, which ultimately did not affect the outcomes related to the diagnosis of chest injuries. Our results highlight that lactate dehydrogenase (LDH) levels at admission are significantly associated with the differential diagnosis of chest injuries in cases of severe polytrauma.

Given the limited sample size of our study, it was not feasible to perform subgroup analyses for multiple organ injuries comprehensively. Therefore, our laboratory is committed to expanding the sample size in future studies. This expansion will allow us to explore additional biomarkers and potentially introduce new screening indicators for other organ injuries. We trust that our rigorous approach to eliminating confounding factors meets your standards and contributes to the robustness of our findings.

Response to Reviewer #2

1. Thank you very much for your insightful comments and for highlighting the limitations of our study. In our analysis, the categorization of chest injuries included various conditions such as lung contusion, rib fractures, sternal fractures, pneumothorax, among others. Due to the limited sample size, detailed subgroup analyses within these categories were not feasible. Consequently, we grouped these conditions under a general category of "chest injuries," defined as chest AIS (Abbreviated Injury Scale) ≥ 1.

As a result, our study primarily demonstrates the utility of lactate dehydrogenase (LDH) as a diagnostic biomarker for detecting chest injuries in patients with severe polytrauma. We acknowledge, as you pointed out, that this classification approach limits our study’s implications for enhancing specific treatment strategies for distinct injuries such as rib fractures or lung contusions. However, the accessibility and rapidity of LDH testing can significantly aid prehospital diagnosis. This is particularly beneficial in scenarios where immediate access to well-equipped medical facilities is not possible or during large public events that necessitate quick triage of patients.

We have also taken your advice into consideration and have included a detailed description of these limitations in the Limitations section of our manuscript. We appreciate your rigorous review and valuable suggestions, which have undoubtedly improved the manuscript.

2. Thank you very much for your suggestion regarding the inclusion of chest X-ray (CXR) as part of the screening criteria. Indeed, in our study, CXR was utilized as one of the foundational diagnostic tools for patients with chest injuries. We acknowledge that incorporating CXR into the screening protocol can enhance the accuracy of injury detection.

However, the primary focus of our study was to evaluate the effectiveness of a rapid hematological screening method as a standalone diagnostic tool, particularly in scenarios where radiological facilities, such as CXR, are not readily accessible—such as at the scene of a natural disaster. The intent was to provide a feasible alternative for initial assessment in severe polytrauma patients, both with and without apparent chest injuries, when traditional radiological diagnostics are unavailable.

Therefore, while CXR results were considered in the patient assessment, our manuscript emphasizes the utility and applicability of hematological screening in resource-limited settings. We believe this focus is crucial for advancing pre-hospital care in less equipped environments, and it complements existing diagnostic protocols that rely on imaging technologies.

We are deeply grateful for the detailed and constructive feedback provided by the reviewers and the editor. Your insights have significantly contributed to refining our manuscript and ensuring that it meets the rigorous standards of PlosOne. We have endeavored to address all the points raised in a comprehensive manner and have made revisions that we believe enhance the value and clarity of our work.

We hope that the changes we have implemented are satisfactory and meet the expectations of the review committee. We are committed to making any further modifications if needed and are looking forward to your suggestions and final decision.

Thank you once again for your guidance and support throughout the review process. We appreciate the opportunity to contribute to PlosOne and eagerly anticipate the possibility of our work being published.

Sincerely yours,

Weining Yan
