# Supplementary material for: Lactate dehydrogenase can be used for differential diagnosis to identify patients with severe polytrauma with or without chest injury—A retrospective study
Source: PLoS One. 2024 Aug 1;19(8):e0308228. doi: 10.1371/journal.pone.0308228 (PMC11293635; doi:10.1371/journal.pone.0308228)
Supplement: Attachment — Submitted filename: Response to Reviewers.docx [file pone.0308228.s004.docx]

Dear Editor and Reviewers,

We sincerely appreciate the time and effort you have invested in reviewing our manuscript titled "Lactate dehydrogenase can be used for differential diagnosis to identify patients with severe polytrauma with or without chest injury—a retrospective study." Your insightful comments and constructive feedback have been invaluable in refining and improving the quality of our work.

Following your recommendations from the initial review, we have undertaken a thorough revision of our manuscript. Your detailed and thoughtful suggestions have significantly contributed to the enhancement of the clarity, robustness, and overall scientific merit of our study. We are deeply grateful for your guidance.

In this second round of revisions, we have meticulously addressed each of the points you raised. We believe the changes we have implemented strengthen the manuscript and align it more closely with the high standards of PlosOne. We have summarized our responses to each comment below, detailing the modifications made to the manuscript to address your concerns.

Thank you once again for providing us with this invaluable opportunity to further improve our work. We hope that the revised manuscript meets your expectations and the esteemed standards of PlosOne.

Response to Reviewer #1

Thank you very much for your insightful comments. As you rightly pointed out, the original description was overly cumbersome. In accordance with your suggestion, I have retained only the definition of 'Severe polytrauma' and have revised the description of the inclusion criteria (lines 66, 79-81). I trust these modifications address your concerns and enhance the clarity and precision of the manuscript.

Response to Reviewer #2

1. Thank you for your constructive feedback regarding the need for a deeper analysis of the sources of elevated LDH levels, which could indeed enhance the credibility and practical value of our study. In response to your suggestion, we conducted a detailed examination of the specific injuries contributing to elevated LDH levels. Given the complexity of these diagnoses, we categorized the injuries as follows: fractures of the chest wall (including ribs, sternum, and thoracic vertebrae), extra-thoracic wall fractures (scapula and clavicle), pulmonary contusion, pleural diseases (pneumothorax, haemothorax, pleural effusion), pneumonia, and cardiovascular diseases (aortic dissection, myocardial contusion, pericardial effusion, mediastinal haematoma).

Our results indicate significant associations between elevated LDH levels and the following conditions: fractures of the chest wall (p=0.001), pulmonary contusion (p<0.001), and cardiovascular diseases (p=0.035). These findings provide valuable guidelines for directing further diagnostic examinations in patients.

In our manuscript, we have elaborated on the significance of these findings and discussed the limitations arising from the low incidence of certain conditions, such as myocardial contusion and aortic coarctation, which prevented a more detailed disease-specific analysis. These changes have been incorporated into lines 198-211 and 260-269 of the revised manuscript. I trust these amendments address your concerns and contribute positively to the manuscript.

2. Thank you for your suggestion regarding the use of pulse oximetry and ultrasound in our study. Our investigation did include an analysis of the partial pressure of blood oxygen; however, we found that it does not reliably indicate chest injuries. This may be due to its insensitivity to injuries that do not directly impact ventilation and gas exchange, noting that chest injuries can extend beyond the lungs and airways.

While we agree that comprehensive diagnostic tools like ultrasound or X-ray can enhance diagnostic accuracy, the primary goal of our study was to develop a rapid screening method for chest injuries in polytrauma patients. This approach is intended to streamline patient triage and optimize the allocation of healthcare resources. LDH testing, which can be performed concurrently with routine blood gas analyses, offers a quick and readily available diagnostic measure without the need for additional equipment or specialized personnel such as ultrasonographers.

We have detailed the benefits of using LDH as a diagnostic tool in the sections on lines 48-53, 252-258, and 266-275 of our manuscript. These sections explain how LDH integrates into clinical protocols to provide effective, efficient care in emergency settings. I appreciate your comments and hope that the revisions and clarifications meet the article's aims and your expectations.

3. Thank you for your insightful comments. As you noted, our study is a preliminary investigation with a limited sample size. Despite these constraints, our methodology—which includes controlling for confounders—has successfully demonstrated a rapid screening technique for chest injuries. This method also aims to guide clinical examinations and optimize the allocation of healthcare resources effectively.

However, we acknowledge several limitations, particularly concerning the specificity of our screening for individual organs and distinct pathologies. In response to these limitations and guided by your valuable suggestions, our laboratory is committed to expanding the sample size in future studies. This expansion will enable us to explore additional biomarkers and potentially develop new screening metrics for other organ injuries.

We appreciate your guidance, which has been instrumental in refining our research approach. We trust that the revisions and future study directions outlined here meet your expectations and contribute meaningfully to the field.

We extend our deepest gratitude to the reviewers and the editor for their detailed and constructive feedback. Your insights have been instrumental in refining our manuscript and ensuring it adheres to the rigorous standards of PlosOne. We have diligently addressed all the points raised, implementing revisions that we believe significantly enhance the clarity, robustness, and overall value of our work.

We sincerely hope that the changes we have made meet your expectations and are satisfactory to the review committee. We remain fully committed to making any further modifications if needed and greatly value your continued guidance and support.

Thank you once again for this opportunity to improve our manuscript. We appreciate the chance to contribute to PlosOne and eagerly anticipate the possibility of our work being published.

Sincerely yours,

Weining Yan
