# Supplementary material for: Lactate dehydrogenase can be used for differential diagnosis to identify patients with severe polytrauma with or without chest injury—A retrospective study
Source: PLoS One. 2024 Aug 1;19(8):e0308228. doi: 10.1371/journal.pone.0308228 (PMC11293635; doi:10.1371/journal.pone.0308228)
Supplement: Attachment — Submitted filename: Response to Reviewers.docx [file pone.0308228.s005.docx]

Dear Reviewers and Editor,

We would like to express our sincere gratitude for the continued time and effort you have dedicated to reviewing our manuscript titled "Lactate dehydrogenase can be used for differential diagnosis to identify patients with severe polytrauma with or without chest injury—a retrospective study." Your ongoing insightful comments and valuable suggestions have been crucial in further refining and enhancing the quality of our work.

Following your detailed feedback from the second review, we have once again undertaken a revision of our manuscript. We greatly appreciate the constructive guidance provided, which has significantly contributed to the robustness and clarity of our study. Each point raised has been carefully considered, and corresponding revisions have been made to address these concerns thoroughly.

We are resubmitting our manuscript after incorporating these additional changes and hope that the revised version meets the high standards of PlosOne. If you believe that incorporating our detailed responses directly into the body of the manuscript would be beneficial, we would be more than happy to do so. Below, we provide detailed responses to each of the comments and outline the modifications made to the manuscript. Thank you once again for this invaluable opportunity to improve our work.

**Responses to Reviewer Comments**

**1. Rationalization of LDH as a Diagnostic Tool in Acute Trauma**

Thank you for your detailed and insightful comments regarding the limitations of LDH as a diagnostic reference marker in well-resourced medical environments. We fully acknowledge that our study has limited utility in guiding the diagnosis and treatment of specific diseases under such conditions. However, the primary aim of our research was to facilitate the rapid screening of polytrauma patients for chest injuries, thereby providing a basis for the necessity of further chest examinations.

In scenarios where medical resources are severely constrained—such as during natural disasters (earthquakes, tsunamis), conflicts, or widespread epidemics—conducting comprehensive examinations on all potentially injured patients within a short timeframe is unfeasible. In these situations, any tool that aids in patient triage can significantly improve the allocation of medical resources and potentially save lives. Thus, our study highlights the utility of LDH in identifying patients with potential chest injuries, allowing healthcare providers to prioritize their evaluations more effectively during periods of resource scarcity, as detailed in lines 252-258 of the manuscript.

Regarding your concern that the use of LDH may deter some imaging tests, we believe our findings will instead assist physicians in determining when imaging is warranted. For instance, a patient with an LDH level exceeding 365 U/L would prompt increased vigilance for chest injuries, thereby reducing the risk of missed diagnoses, even if the primary injury is elsewhere, such as an open fracture of the lower extremity with hemorrhagic shock.

Moreover, our study aimed to identify patients who are "more likely" to have chest injuries, allowing for prioritized treatment and avoiding unnecessary delays for those less likely to have such injuries. This stratification is crucial for the rational allocation of limited healthcare resources, enabling more efficient and targeted patient care.

We hope this clarifies the scope and significance of our study in contexts with limited medical resources.

**2. Clarification on Aortic Dissection**

Thank you for your correction. We indeed referred to aortic tears resulting from trauma. We have updated the description in the manuscript to "blunt aortic injury."

**3. LDH as a Cell Necrosis Marker**

Thank you for your insightful comments. Lactate dehydrogenase (LDH) is recognized as a marker of cellular necrosis, and our findings suggest that it can be effectively used for the differential diagnosis of chest injuries in polytrauma patients. However, we did not evaluate LDH in comparison to other markers of cellular necrosis, such as TNFα, because these are not commonly assessed in routine clinical practice. This approach allows our results to be more directly applicable to everyday clinical settings.

While it is acknowledged that LDH lacks tissue specificity, displaying only a 1.5-fold difference in activity between tissues with the highest (e.g., liver) and lowest (e.g., kidney) LDH levels, our study still observed elevated LDH levels in patients with chest injuries, despite having similar overall trauma severity scores (ISS=30.43 ± 9.28 vs. 30.88 ± 10.24, P=0.894). This elevation likely reflects the involvement of organs within the chest that are critical to respiratory and circulatory functions, resulting in greater cellular necrosis and subsequent release of LDH into the bloodstream.

We have detailed this rationale in lines 237-252 of the manuscript. If LDH can demonstrate significant differences between groups, the utility of other markers, even if potentially more specific, becomes less critical in the context of routine clinical assessments. We hope this clarification meets your requirements and enhances the manuscript.

**4. Comparison with Troponin**

Thank you for your insights regarding the use of cardiac troponins (cTnI and cTnT) as specific markers of myocardial injury. We recognize that troponins are superior to lactate dehydrogenase (LDH) in identifying cardiac damage. However, the primary diagnostic aim of our study was not solely to detect cardiac injury but to ascertain the presence of any chest injury in polytrauma patients. Since the chest includes a variety of tissues, such as the lungs and chest wall, normal troponin levels do not necessarily exclude the presence of non-cardiac chest injuries. Our results support the utility of LDH as a broader marker in this context, providing a simple and routinely available diagnostic option that can indicate a range of chest injuries during initial patient evaluations.

Additionally, we also analyzed creatine kinase (CK) and CK-MB in our study. Despite the high cardiac specificity of CK-MB, it did not show potential in identifying general chest injuries among polytrauma patients, further highlighting LDH’s broader applicability for this purpose. These findings reinforce the importance of LDH in the initial assessment of polytrauma patients, where a comprehensive evaluation of all possible chest injuries is critical.

We trust this explanation clarifies the rationale behind our focus on LDH and its role in our study. We appreciate your valuable feedback and hope that our findings contribute meaningfully to the field.

**5. Positive and Negative Predictive Values**

Thank you for your pertinent and insightful comments. It is generally accepted that NPV and PPV values above 80% are ideal for clinical diagnosis. While our results are very close to this threshold, we acknowledge that the diagnosis of specific chest injuries ultimately relies on targeted tests such as ultrasound, CT, and other imaging modalities.

The significance of our study lies in the potential of LDH to aid physicians in pre-screening patients who are likely to have chest injuries. This preliminary screening can guide clinicians to perform more targeted and efficient examinations, optimizing the use of diagnostic resources and potentially improving patient outcomes.

**6. Addressing Type 2 Error**

Thank you for your attention to the details of our study. We validated our data through efficacy analysis. In our binary logistic regression analysis, we achieved an acceptable result with N=64, α=0.05, and a power of 0.83 (a power of 0.8 is usually acceptable in scientific research). We have prepared the test report in PDF format and are ready to provide it for your review. Please advise on the preferred method for submission if submission is required, as the manuscript submission guidelines do not specify where such documents should be uploaded or sent.

**7. Specificity to Chest Injury**

As discussed in response to question No. 3, our statistical analysis clearly demonstrates that elevated LDH levels correlate significantly with additional chest trauma in severely injured patients. This correlation is mathematically significant, reinforcing LDH's utility as a biomarker in this context.

Although LDH is not tissue-specific—studies indicate only about a 1.5-fold difference between the tissues with the highest LDH values (e.g., liver) and those with the lowest (e.g., kidney)—we observed a notable increase in LDH levels in patients with chest injuries, despite comparable overall trauma levels (ISS=30.43 ± 9.28 vs. 30.88 ± 10.24, P=0.894). This observation may be attributed to the involvement of organs within the chest that are essential to the respiratory and circulatory systems, which could lead to increased cellular necrosis and subsequent LDH release into the bloodstream, as detailed in lines 237-252 of the manuscript.

It is important to recognize that elevated LDH levels in patients with chest injuries may not solely arise from the factors described. The underlying mechanisms remain a subject for further investigation, a direction we have committed to pursuing in future research, as outlined in our article.

**8. Influence of Treatment Factors on LDH**

Thank you for your suggestion. In our analysis, we considered the effect of blood transfusion by examining the transfusion of red cells, plasma, and platelets in patients with and without chest injuries. Our results, presented in S1 Table, indicate no significant difference between the groups.

Surgery may indeed affect LDH levels, which could explain why the between-group differences in LDH became non-significant at subsequent observation time points. However, our findings demonstrate that LDH levels at admission can be used to identify the presence of chest injury in polytrauma patients. At the time of admission, none of the patients had undergone surgery, so the LDH levels were not influenced by surgical intervention.

**9. Addition to Existing Diagnostic Methods**

Thank you for your insights regarding the reliability of using elevated LDH levels as a sole diagnostic marker. I concur that a definitive diagnosis of specific diseases based solely on LDH levels would be unreliable. However, our findings suggest that LDH can serve effectively as an indicative marker for potential concomitant chest injuries in polytrauma patients. It is important to clarify that our study does not advocate for the abandonment of established diagnostic tests such as X-rays, CT scans, and ultrasounds; rather, we propose that LDH can be used to guide more targeted diagnostic efforts.

To prevent any potential misunderstandings, we have included a clarifying note in lines 266-268 of the manuscript, emphasizing that LDH should not replace more comprehensive diagnostic methods like ultrasound or CT scans. These tools are indispensable for confirming disease diagnoses and monitoring disease progression.

In resource-constrained settings, such as during natural disasters or conflicts, the ability to quickly classify patients using basic laboratory tests like LDH can be invaluable. This method enables a more efficient allocation of limited medical resources by prioritizing the examination of patients most likely to have significant injuries. Additionally, detecting elevated LDH levels can raise awareness among physicians of possible underlying injuries that may not be immediately apparent from the patient's primary complaints.

We hope this explanation addresses your concerns and clarifies the intent and scope of our research.

We are deeply grateful for the detailed and constructive feedback provided by the reviewers and the editor throughout this review process. Your insights have been instrumental in refining our manuscript and ensuring it meets the rigorous standards of PlosOne. We have endeavored to address all the points raised in a comprehensive manner, and we believe the revisions we have implemented significantly enhance the value and clarity of our work.

We sincerely hope that the changes made are satisfactory and meet the expectations of the review committee. Should you find it advantageous for our responses to be integrated into the manuscript itself, we are more than willing to make such modifications. We remain committed to making any further adjustments if needed and greatly value your continued guidance and support.

Thank you once again for your assistance and for providing us with this opportunity to improve our manuscript. We appreciate the chance to contribute to PlosOne and eagerly anticipate the possibility of our work being published.

Sincerely yours,

Weining Yan
